# Association between loneliness and mental health among nurses: a cross-sectional research in China

**DOI:** 10.1590/1414-431X2024e13408

**Published:** 2024-07-01

**Authors:** Yu Qiao, Chao Wang, Xueyan Tian, Ming Cao

**Affiliations:** 1 Department of Operating Room, Shengjing Hospital of China Medical University, Shenyang, China; 2 Department of Urology, Shengjing Hospital of China Medical University, Shenyang, China

**Keywords:** Loneliness, Depression, Anxiety, Coronavirus disease 2019 (COVID-19), Nurses, China

## Abstract

This study explored the association between loneliness and mental health among nurses in China during the COVID-19 pandemic. This cross-sectional study was conducted from March to April 2022. We enrolled 2,811 nurses from a tertiary hospital in China. Demographic characteristics, lifestyle factors, work-related factors, and psychological characteristics were collected from participants via a self-reported questionnaire. Loneliness was measured with the three-item short form of the Revised UCLA Loneliness Scale, and the Patient Health Questionnaire (PHQ-9) and the General Anxiety Disorder (GAD-7) scale were used to measure mental health. Adjusted odds ratios (ORs) and 95% confidence intervals (CI) were determined using binary logistic regression. Among participants in this study, 12.0% (337) experienced loneliness, and 7.8% (219) and 6.7% (189) reported depression and anxiety, respectively. The loneliness scores were categorized into three levels (3, 4-6, and 7-9). For depression, compared with the lowest reference, the ORs and 95% CI across the tertile were 1.31 (0.69-1.84) and 2.53 (1.11-5.76) after adjustment, respectively, and the P-value for trend was 0.045. For anxiety, compared with the lowest reference, the ORs and 95%CI across the tertile were 1.84 (1.28-2.63) and 2.52 (1.57-4.10) after adjustment, respectively; the P-value for trend was 0.004. This study showed that loneliness was significantly associated with poor mental health among nurses during the COVID-19 pandemic. These findings suggested that medical establishments should offer interventions for nurses to prevent mental health problems by targeting this modifiable risk factor.

## Introduction

The 2019 Global Burden of Diseases report showed that the two most common mental disorders were depression and anxiety (1). Depression is a prevalent disorder with persistent low mood, diminished interest, and loss of pleasure (2). It is a common illness worldwide, with an estimated 3.8% of the population affected, including 5.0% of adults (3). Anxiety is a condition characterized by fear, anxiousness, distress, and perceived environmental threat (4). It has a significant impact on society, with a prevalence rate of 3.3% among the general population (5). People who experience chronic or recurrent attacks of depression or anxiety suffer from poor performance at work and disrupted family life. At its worst, depression could lead to suicide (6). A study from 204 countries and territories around the world in 2020 found a substantial increase in the prevalence of major depression and anxiety during the COVID-19 pandemic (7). A review among healthcare workers showed that the prevalence of anxiety and depression during the pandemic was as high as 24.94 and 24.83%, respectively (8).

Loneliness is defined as a cognitive discrepancy between desired and experienced social relationships (9). It has been recognized as a significant public health concern that is detrimental to mental and social health throughout life, influencing outcomes such as suicidal ideation (10). It is an independent risk factor for many chronic diseases such as cardiovascular diseases and obesity (11). During the COVID-19 pandemic, loneliness became a critical public health concern affected by various factors, such as working at home, shelter-in-place, avoiding people gatherings, severe social isolation, and quarantine restrictions (12). A survey of 15,530 respondents in the UK showed high prevalence rates of loneliness (35.86%) during the COVID-19 pandemic (13).

Numerous previous studies have demonstrated an association between loneliness and poor mental health, especially among older adults. However, less research has been conducted among young adults or the middle-aged population who are actively working. In a cross-sectional online survey including 1,421 healthcare professionals in Spain, Cabello et al. (14) found that loneliness was positively related to mental health problems during the COVID-19 pandemic. A longitudinal study of 458 university students found a reciprocal association between loneliness and depression, and loneliness could predict anxiety across time. In addition, no gender differences were observed in the subgroup analysis (15). A recent multi-center, cross-sectional study including 117 nurses showed that loneliness had a positive association with burnout, and the authors concluded that loneliness could be a contributor to burnout (16).

As an essential part of the healthcare workforce, nurses account for the high occupational burden during the COVID-19 pandemic (17,18). A study conducted in China showed that nurses experienced more unfavorable mental health outcomes than other healthcare workers during the COVID-19 pandemic (19). Moreover, poor mental health influences the nurses’ professional performance, the quality of the health care provided, and the safety of patients (20,21); however, there is limited data for this group of workers and no data about the association between loneliness and anxiety and depression. Therefore, we conducted a large cross-sectional study to explore whether there is an association between loneliness and mental health (anxiety and depression) among nurses in China during the COVID-19 pandemic.

## Material and Methods

### Study design

**Figure 1:**
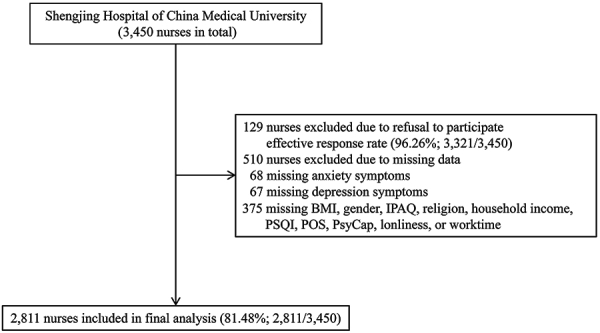
Flowchart of participant recruitment. BMI: body mass index; IPAQ: International Physical Activity Questionnaire; PSQI: Pittsburgh Sleep Quality Index; POS: Perceived Organization Support; PsyCap: Psychological Capital.

Between March and April 2022, the nursing department of the Shengjing Hospital of China Medical University conducted this cross-sectional study. It is a tertiary hospital with more than 6,700 beds. From a total of 3,450 nurses working in this hospital who took part in the present study, effective responses were obtained from 2,811 individuals (effective response rate: 81.48%). A set of self-administered questionnaires was adopted. Participants completed a structured questionnaire within 20 to 25 min. Figure 1 shows a flowchart outlining the procedure.

The Ethics Committee of Shengjing Hospital Affiliated China Medical University in Shenyang, China provided ethical approval (#2022PS753K). All participants gave written consent after being fully informed. The interventions were carried out in accordance with the 1975 Declaration of Helsinki's ethical guidelines.

### Inclusion and exclusion criteria

The following qualifications were required for inclusion: being a working nurse at a hospital. Nurses who had been employed for less than three months or who did not submit all requested information on the questionnaire were not included.

### Measurement of characteristics

Age, gender, and body mass index (BMI, kg/m^2^) were among the demographic variables of this study. Smoking status, alcohol use, and coffee consumption were all considered to be lifestyle factors. Life-related factors included sleep quality (PSQI, Pittsburgh Sleep Quality Index scores), physical activity (IPAQ, International Physical Activity Questionnaire, metabolic equivalent (METS) × hour/week), religion, marital status, siblings, monthly household income (RMB, yuan, categorized as, <5,000, ≥5,000 <10,000, and ≥10,000), major life events, and history of chronic disease. Years of employment, specialty (surgery, internal medicine, obstetrics and gynecology, pediatrics, etc.), working hours per week, and night shifts were all work-related factors.

Smoking behavior was divided into three categories: current smokers (more than one cigarette per day for the past six months), former smokers (who haven't smoked in six months), and never smokers. Alcohol and coffee consumption patterns were broken down into three categories: current drinkers (one time per day for the past six months), former drinkers (who stopped drinking at least six months before), and never drinkers. Major life events that were experienced included separation or divorce, death or serious illness of close family members, severe injury or traffic accidents, violence (including workplace violence, such as insults, threats, and physical attacks), unemployment, natural disasters, death or serious illness of a partner, serious conflict with family, medical disputes, and income reduction or debt. History of physical illness was evaluated based on response (“Yes” or “No”) to questions concerning a history of diseases. Nurses who interacted with COVID-19 suspected or positive patients or came into contact with circumstances requiring COVID-19 quarantine were considered exposed to the COVID-19 pandemic.

Using the short form of the International Physical Activity Questionnaire, physical activity over the previous week was evaluated (22). The questionnaire asked whether participants had performed any activities from the following categories during the previous week: walking, moderate activity (household activity or child care), or vigorous activity (running, swimming, or other sports activities). We calculated the MET hours per week using coefficients that corresponded to the activity categories (3.3, 4.0, and 8.0, respectively) according to the following formula: MET = coefficient of activity × duration (hours) × frequency (days). Total physical activity level was determined by combining separate scores for each activity performed in the previous week.

The PSQI was used to measure sleep quality (23). Participants self-reported their sleep quality over the last 4 weeks. The first 4 items looked at times (e.g. bed time, number of minutes it took to fall asleep, wake up time, hours of sleep per night). The next 10 items asked how often the participant had trouble sleeping because of different reasons (e.g. woke up in the middle of the night, needed to go to the bathroom, coughed, had a bad dream). Each of these questions was answered on a 4-point scale ranging from “never” to “3 times or more a week”. Three questions asked for a subjective rating of the participant's sleep quality (4-point scale from “very good” to “very bad”), use of sleep medication, and if the participant had trouble staying awake during the day (4-point scale ranging from “never” to “3 times or more a week”). The final question asked if the participant had trouble keeping up enthusiasm for getting things done (4-point scale ranging from “no problem at all” to “a very big problem”). Responses to these 18 items formed a series of 7 scores (sleep quality, sleep latency, sleep duration, sleep efficiency, sleep disturbances, sleep medication, and daytime dysfunction) that ranged from 0 to 3. These component scores were added to make up a general score. Higher scores represented worse sleep quality. Poor sleep quality was indicated by a total score of 6 or greater.

The degree of organizational support was assessed using the Chinese translation of the Perceived Organization Support (POS) questionnaire (24). For POS, the Cronbach's alpha coefficient was 0.921. The 24-item Psychological Capital Questionnaire (PCQ), which has a validated Chinese translation, was used to assess psychological capital (PsyCap) (25,26). Cronbach's alpha coefficients of self-efficacy, hope, resilience, and optimism were 0.921, 0.936, 0.920, and 0.900, respectively. The three-item short variant of the Revised UCLA Loneliness scale was used to measure loneliness (27). The questions were: “How often do you feel that you lack companionship?”, “How often do you feel left out?”, and “How often do you feel isolated from others?”. The answers for each question were “hardly ever” (1 score), “some of the time” (2 scores), or “often” (3 scores). The cut-off point for loneliness was 6 (3-9), which is consistent with other research (28). PHQ-9 scores were used to measure depressive symptoms (29). A PHQ-9 score of 10 was considered to indicate the presence of severe depression. The General Anxiety Disorder (GAD-7) questionnaire was used to quantify anxiety; a threshold value of 10 was used to identify anxiety (30). For loneliness, PHQ-9, and GAD-7, the Cronbach's alpha coefficients were 0.930, 0.951, and 0.928, respectively.

### Statistical analysis

The data were analyzed using SPSS 22.0 for Windows (SPSS Inc., USA). Continuous variables are reported as median (interquartile range) and categorical variables as number and percentage. The means of two non-normally distributed continuous variables from independent samples were compared using the Mann-Whitney U test. To compare the means of two normally distributed continuous variables, the Student's *t*-test was employed. The Fisher's exact test or the χ^2^ test was used for categorical variables.

Tertiles were categorized across loneliness ratings (3, 4-6, and 7-9) and used for further research based on the distribution for all participants. To investigate relationships between loneliness tertile categories and mental health states (depression and anxiety), binary unconditional logistic regression analysis was utilized. Loneliness score was the independent variable, while mental health was the dependent variable. Before determining the crude OR, the crude model and model 1 were adjusted for age, gender, and BMI. Model 2 was further adjusted for baseline variables that were considered clinically relevant or that had a P-value <0.10 in the univariate analysis, including alcohol use, sleep quality (PSQI scores), siblings, experiences of major life events, worktime duration, and POS and PsyCap scores for depression; and sleep quality, physical activity, marital status, siblings, experiences of major life events, history of chronic disease, years of employment, specialty, worktime duration, and POS and PsyCap scores for anxiety. Model 3 was adjusted for all baseline variables. Adjusted odds ratios and 95% confidence intervals (CI) were computed when relevant covariates were taken into account using binary unconditional logistic regression. In order to check for a linear trend across rising tertiles, the median value of each tertile was employed as a continuous variable. The difference was deemed significant at P<0.05, and all P values were two-tailed.

Because there was 11.6% (370/3,181) of missing data for some confounding variables (excluding exposures and dependent variables) in this study, we performed sensitivity analyses in which we repeated the main analyses after multiple imputation of missing data. We assumed data were missing at random. We did 10 imputations of missing data by chained equations approach method.

## Results

From a total of 3,450 nurses, 2,811 nurses were enrolled in this study, with a median age of 35 years, a median BMI of 21.83 kg/m^2^, and 94.2% of the participants were women. Among participants, 12% (337) experienced loneliness; 7.8% (219) and 6.7% (189) reported depression and anxiety, respectively.

In the univariate analysis, participants with depression had higher BMI, poor sleep quality, and lower scores of perceived organization support. The majority of them had alcohol habits, had siblings, experienced major life events, worked for more than five years, and worked more than 40 h per week. Participants with anxiety were older, had higher BMI, poorer physical activity habits, poorer sleep quality, and lower scores of perceived organization support than those without anxiety. Moreover, the majority of participants were married, had siblings, experienced major life events, had a history of chronic disease, worked for more than five years, worked in surgical departments, and worked more than 40 h per week; see more details in Supplementary Table S1.

In the association between loneliness and depression, compared with the lower tertile of loneliness, the ORs of the middle and upper tertiles were 1.31 (95%CI: 0.69-1.84) and 2.53 (95%CI: 1.11-5.76) after adjustment, respectively, and the P for trend was 0.045. For anxiety, compared with the lower tertile of loneliness, the ORs of the middle and upper tertiles were 1.84 (95%CI: 1.28-2.63) and 1.52 (95%CI: 0.57-4.10) after adjustment, respectively, and the P for trend was 0.004, see details in Table 1.

**Table 1:** Association between loneliness level and mental health.

	Level of loneliness (range, n=2,811)			P for trend^a^
	Level 1 (3)	Level 2 (4-6)	Level 3 (7-9)	
Depression				
No. of participants	1,695	1,077	75	
With depression	102	105	12	
Crude	Reference	1.65 (1.24, 2.19)	2.91 (1.52, 5.56)	<0.001
Adjusted model 1^b^	Reference	1.66 (1.25, 2.20)	2.87 (1.50, 5.51)	<0.001
Adjusted model 2^c^	Reference	1.31 (0.69, 1.84)	**2.53 (1.11, 5.76)**	**0.045**
Adjusted model 3^d^	Reference	1.24 (0.87, 1.78)	**3.56 (1.58, 8.01)**	**0.008**
Anxiety				
No. of participants	1,695	1,077	75	
With anxiety	84	96	9	
Crude	Reference	1.84 (1.36, 2.49)	2.56 (1.23, 5.31)	<0.001
Adjusted model 1^b^	Reference	1.84 (1.36, 2.49	2.25 (1.07, 4.73)	<0.001
Adjusted model 2^c^	Reference	**1.84 (1.28, 2.63)**	**2.52 (1.57, 4.10)**	**0.004**
Adjusted model 3^d^	Reference	**2.03 (1.41, 2.93)**	**2.53 (1.56, 4.22)**	**0.003**

Data are reported as odds ratio (95% confidence interval). ^a^Multiple logistic regression analysis. ^b^Adjusted for age, gender, and body mass index. ^c^Additionally adjusted for alcohol habit, sleep quality, siblings, experiences of major events, worktime duration, and POS and PsyCap scores for depression; and sleep quality, physical activity, marital status, siblings, experiences of major events, history of chronic disease, years of employment, specialty, worktime duration, and POS and PsyCap scores for anxiety based on Model 1. ^d^Additionally adjusted for all baseline variables. Bold type indicates P<0.05.

We repeated our main analyses after multiple imputation of missing data. The results from the sensitivity analyses did not significantly change the estimated associations of the main analyses (Supplementary Table S2).

## Discussion

Undoubtedly, the outbreak of COVID-19 has had a considerable impact on loneliness and mental health. Although an increasing number of studies reported a high incidence of depression and anxiety among healthcare workers (31), there are fewer studies concerning the association between loneliness and mental health, especially in nurses. This study showed that loneliness is positively associated with the development of both depression and anxiety among nurses.

In line with this, a large cross-sectional research in Spain using the GHQ-12 questionnaire, which enrolled 1,421 healthcare professionals and resident physicians, found that loneliness was associated with poor mental health, which could not be categorized as depression and anxiety (14). Furthermore, a longitudinal population-based study on adults aged over 50 years reported loneliness as a risk factor for mental health disorders during the COVID 19 pandemic, compared with the pre-COVID-19 trend (32). One of the possible mechanisms is that loneliness causes a greater self-centered inability to respond to threats and may result in poor mental health through intolerance of uncertainty and insomnia (33). Another possible mechanism is that the increased activity of pro-inflammatory transcription control pathways in individuals who experience loneliness results in a high level of inflammatory cytokine secretion, which plays an essential role in the development of depression (34,35). However, previous studies suggest that the association between loneliness and depression is bidirectional. One cohort study determined that depression was both the outcome and the preceding variable of loneliness. Simultaneously, anxiety tended to be the outcome variable of loneliness (15). This needs to be explored further.

A recent multi-center cross-sectional study including 117 nurses showed that loneliness had a positive association with burnout. The authors concluded that loneliness can contribute to burnout. The study also revealed that nurses have a sense of invisibility, emotional disengagement from their work, and dehumanization. In addition, maintaining social bonds with colleagues serves as a safeguard, as nurses consistently express a deep dedication to their job and a sense of solidarity with their peers (16). Another cross-sectional study including 265 medical employees (69.8% of them were nurses) from psychiatric wards during the COVID-19 pandemic also found that loneliness was a significant predictor of exhaustion and disengagement (36).

The current study had several limitations. First, it was a single-center cross-sectional study; a multi-center prospective cohort study is needed in the future. Second, the subgroup analysis by gender was not possible due to the limited number of male participants (5.8%). Third, the data were based on self-reported questionnaires, which may be affected by recall bias. Fourth, the models could not be adjusted for some potentially important variables, such as educational background and experience with mental health help. Fifth, the GAD-7 and PHQ-9 are screening questionnaires that cannot be used for clinical diagnosis, which may have affected our results. Sixth, the measurement of alcohol intake (times per day) was not accurate and could also have influenced the results.

### Conclusion

This study showed that loneliness was significantly associated with poor mental health among nurses during the COVID-19 pandemic. These findings suggested that medical establishments should offer interventions for nurses to prevent mental health problems by targeting this modifiable risk factor.

## Supplementary Material

Click here to view [pdf].
