# Supplementary material for: Association between loneliness and mental health among nurses: a cross-sectional research in China
Source: Braz J Med Biol Res. 2024 Jul 1;57:e13408. doi: 10.1590/1414-431X2024e13408 (PMC11221866; doi:10.1590/1414-431X2024e13408)
Supplement: Supplementary file 1 [file 1414-431X-bjmbr-57-e13408-suppl.pdf]

Table S1. Baseline characteristics according to mental health (2,811 participants).

Variables	Total	Depression			Anxiety		
		With	Without	P	With	Without	P
Number (%)	2,811	219 (7.80)	2,592 (92.20)		189 (6.70)	2,622 (93.30)	
Demographic characteristics							
Age (years)	35.00 (32.00, 37.00)	35.00 (32.00, 37.00)	35.00 (32.00, 37.00)	0.810	35.00 (33.00, 38.00)	35.00 (32.00, 37.00)	**<0.001**
Gender (female)	2,649 (94.20)	210 (95.90)	2,439 (94.10)	0.277	180 (95.20)	2,469 (94.20)	0.542
BMI (kg/m^2)	21.83 (20.07, 23.88)	22.57 (20.76, 24.38)	21.76 (19.96, 23.80)	**0.002**	22.66 (20.76, 24.09)	21.77 (19.95, 23.83)	**0.005**
Lifestyle factors							
Smoking habit				0.659			0.431
Current	36 (1.30)	3 (1.40)	33 (1.30)		3 (1.60)	33 (1.30)	
Former	24 (0.90)	3 (1.40)	21 (0.80)		3 (1.60)	21 (0.80)	
Never	2,751 (97.90)	231 (97.30)	2,538 (97.90)		183 (96.80)	2,568 (97.90)	
Alcohol habit				**0.039**			0.624
Current	192 (6.80)	24 (11.00)	168 (6.50)		15 (7.90)	177 (6.80)	
Former	144 (5.10)	12 (5.10)	144 (5.40)		9 (4.80)	135 (5.10)	
Never	2,475 (88.00)	198 (83.50)	2,355 (88.00)		165 (88.10)	2,310 (88.10)	
Coffee habit				0.606			0.621
Current	768 (27.30)	60 (27.40)	708 (27.30)		45 (23.80)	723 (27.60)	
Former	423 (15.00)	39 (17.80)	384 (14.80)		36 (19.00)	387 (14.80)	
Never	1,620 (57.60)	120 (54.80)	1,500 (57.90)		108 (57.10)	1,512 (57.70)	
Sleep quality (PSQI scores)	5.00 (3.00, 8.00)	10.00 (8.00, 12.00)	5.00 (3.00, 7.00)	**<0.001**	10.00 (7.00, 12.00)	5.00 (3.00, 7.00)	**<0.001**
Physical activity (IPAQ Mets×hour/week)	18.60 (3.65, 52.80)	14.60 (0.00, 46.20)	19.30 (4.00, 53.77)	0.824	20.50 (0.00, 87.10)	18.39 (4.00, 51.30)	**0.006**
Have religion (yes), n (%)	93 (3.30)	90 (3.50)	3 (1.40)	0.107	3 (1.60)	90 (3.40)	0.182
Marital status				0.117			**0.099**
Single	543 (19.30)	48 (21.90)	495 (19.10)		30 (15.90)	513 (19.60)	
Married/cohabitation	2220 (79.00)	171 (78.10)	2049 (79.10)		153 (81.00)	2,067 (78.80)	
Divorce/separation/widow	48 (1.70)	0 (0.00)	48 (1.90)		6 (3.20)	42 (1.60)	
Have siblings (yes)	2,013 (71.60)	168 (76.70)	1,845 (71.20)	**0.082**	150 (79.40)	1,863 (71.10)	**0.015**
Household income (Yuan/month)				0.905			0.258
<5,000	15 (0.50)	3 (1.40)	12 (0.50)		0 (0.00)	15 (0.60)	
≤5,000 to <10,000	462 (16.40)	33 (15.10)	429 (16.60)		39 (20.60)	423 (16.10)	
≥10,000	2,334 (83.0)	183 (83.60)	2,151 (83.00)		150 (79.40)	2,184 (83.30)	
Experienced major events (yes)	1,443 (51.30)	147 (67.10)	1,296 (50.00)	**<0.001**	117 (61.90)	1,326 (50.60)	**0.003**
History of chronic disease (yes)	522 (18.60)	42 (19.20)	480 (18.50)	0.810	45 (23.80)	477 (18.20)	**0.056**
Work related factors							
Years of employment				0.100			**0.067**
<5 years	384 (13.70)	18 (8.20)	366 (14.10)		12 (6.30)	372 (14.20)	
5–10 years	1,137 (40.40)	96 (43.80)	1,041 (40.20)		87 (46.00)	1,050 (40.00)	
>10 years	1,290 (45.90)	105 (47.90)	1,185 (45.70)		90 (47.60)	1,200 (45.80)	
Specialty				0.270			**0.004**
Surgery	1,209 (43.00)	105 (47.90)	1,104 (42.60)		120 (63.50)	1,089 (41.50)	
Internal medicine and others	321 (11.40)	24 (11.00)	297 (11.50)		18 (9.50)	303 (11.60)	
Obstetrics and Gynecology	342 (12.20)	15 (6.80)	327 (12.60)		6 (3.20)	336 (12.80)	

Pediatrics	255 (9.10)	6 (2.70)	249 (9.60)		3 (1.60)	252 (9.60)	
Others	684 (24.30)	69 (31.50)	615 (23.70)		42 (22.20)	642 (24.50)	
Worktime duration (hours/week)				**<0.001**			**<0.001**
<40 h	1,758 (62.50)	63 (28.80)	897 (34.60)		54 (28.60)	906 (34.60)	
40–60 h	960 (34.20)	135 (61.60)	1,623 (62.60)		114 (60.30)	1,644 (62.70)	
>60 h	93 (3.30)	21 (9.60)	72 (2.80)		21 (11.10)	72 (2.70)	
Night shifts (more than 3 times/month)	1,590 (56.60)	135 (61.60)	1,455 (56.10)	0.115	117 (61.90)	1,473 (56.20)	0.126
Exposure to the COVID-19 (yes)	315 (11.20)	30 (13.60)	285 (11.00)	0.135	24 (12.70)	291 (11.10)	0.160
Psychological characteristics							
Loneliness (score)	3.00 (3.00, 5.00)	4.00 (3.00, 5.00)	3.00 (3.00, 5.00)	**<0.001**	4.00 (3.00, 5.00)	3.00 (3.00, 5.00)	**<0.001**
Loneliness (yes)	337 (12.00)	34 (15.5)	303 (11.7)	**0.093**	33 (17.5)	304 (11.6)	**0.016**
POS scores	51.00 (44.00, 57.00)	44.00 (36.00, 50.00)	51.00 (45.00, 57.00)	**<0.001**	46.00 (40.00, 51.00)	51.00 (45.00, 57.00)	**<0.001**
PsyCap-efficacy (score)	29.00 (24.00, 31.00)	24.00 (21.00, 26.00)	30.00 (24.14, 32.75)	**<0.001**	24.00 (21.00, 28.00)	30.00 (24.00, 32.00)	**<0.001**
PsyCap-hope (score)	30.00 (24.00, 32.00)	23.69 (21.00, 26.00)	30.00 (25.00, 32.00)	**<0.001**	24.00 (20.00, 29.00)	30.00 (24.00, 32.00)	**<0.001**
PsyCap-resiliency (score)	27.00 (24.00, 31.00)	24.00 (21.51, 27.00)	27.00 (24.00, 31.00)	**<0.001**	25.00 (23.00, 27.00)	27.00 (24.00, 31.00)	**<0.001**
PsyCap-optimism (score)	26.00 (23.00, 28.00)	23.00 (22.00, 25.00)	26.00 (24.00, 29.00)	**<0.001**	23.00 (22.00, 24.00)	26.00 (24.00, 29.00)	**<0.001**

Continuous variables are reported as median (interquartile range) and categorical variables are reported as number (percentage). Student's *t*-test was used to compare the mean of two continuous normally distributed variables, and the Mann-Whitney U test was used to compare the mean of two continuous non-normally distributed variables. The chi-squared test or Fisher's exact test was used for categorical variables. Bold type indicates P<0.05. BMI: body mass index; PSQI: Pittsburgh Sleep Quality Index; IPAQ: International Physical Activity Questionnaire; COVID-19: Coronavirus Disease 2019; POS: Perceived Organization Support; PsyCap: Psychological Capital.

Table S2. Association between loneliness level and mental health of nurses after multiple imputation of missing data.

	Level of loneliness (range, n=3,181)			P for trend[a]
	Level 1 (3)	Level 2 (4–6)	Level 3 (7–9)	
Depression				
No. of participants	1,756	1,111	73	
With depression	93	110	11	
Crude	Reference	1.68 (1.30, 2.17)	2.93 (1.60, 5.35)	<0.001
Adjusted model 1[b]	Reference	1.69 (1.30, 2.20)	2.91 (1.59, 5.33)	<0.001
Adjusted model 2[c]	Reference	1.36 (0.99, 1.86)	**2.52 (1.20, 5.30)**	**0.009**
Adjusted model 3[d]	Reference	1.31 (0.95, 1.81)	**3.11 (1.48, 6.56)**	**0.006**
Anxiety				
No. of participants	1,783	1,111	73	
With anxiety	93	110	11	
Crude	Reference	1.90 (1.43, 2.53)	2.89 (1.48, 5.63)	<0.001
Adjusted model 1[b]	Reference	1.88 (1.41, 2.50)	2.62 (1.34, 5.13)	<0.001
Adjusted model 2[c]	Reference	**1.92 (1.36, 2.72)**	**2.24 (1.07, 5.55)**	**0.001**
Adjusted model 3[d]	Reference	**2.03 (1.41, 2.93)**	**2.53 (1.56, 4.22)**	**0.001**

Data are reported as odds ratio (95% confidence interval). [a]Multiple logistic regression analysis. [b]Adjusted for age, gender, and body mass index. [c]Additionally adjusted for alcohol habit, sleep quality, siblings, experiences of major events, worktime duration, and POS and PsyCap scores for depression; and sleep quality, physical activity, marital status, siblings, experiences of major events, history of chronic disease, years of employment, specialty, worktime duration, and POS and PsyCap scores for anxiety based on Model 1. [d]Additionally adjusted for all baseline variables. Bold type indicates P<0.05.
